# Notch2 Deletion Compromises Epithelial Integrity and Enamel Formation in Rodent Incisors

**DOI:** 10.3390/cells14151224

**Published:** 2025-08-07

**Authors:** Argyro Lamprou, Cristina Porcheri, Thimios A. Mitsiadis

**Affiliations:** 1 Institute of Oral Biology, Centre of Dental Medicine, Faculty of Medicine, University of Zurich, 8032 Zurich, Switzerland; cristina.porcheri@zzm.uzh.ch; 2 Foundation for Research and Technology-Hellas (FORTH), University of Crete, 700 13 Heraklion, Greece

**Keywords:** tooth, Notch signalling, Notch2, cell fates, rodent incisors, dental epithelium, enamel, intercellular junctions, transgenic mice

## Abstract

The evolutionarily conserved Notch signalling pathway regulates the fate, proliferation and differentiation of cells in most developing organs, thus affecting their morphogenesis and function. Here, we investigated the role of the Notch2 receptor in the generation and function of epithelial cells of the continuously erupting rodent incisors. We used transgenic *Notch1*-Cre^ERT2/+^;Rosa26^mT/mG^ and *Notch2*-Cre^ERT2/+^;Rosa26^mT/mG^ mice to compare the contribution of *Notch1-* and *Notch2-*expressing cells and their progeny in the generation of the different epithelial cell populations. Furthermore, we examined if the dental epithelium organisation and enamel structure are affected in early postnatal incisors of *Keratin14*^Cre/+^;*Notch2*^fl/fl^ mice using immunofluorescent staining, gene expression analysis, microcomputed tomography and scanning electron microscopy. Our results showed that Notch2 deletion resulted in smaller incisors with disorganised dental epithelium and defective enamel. Delayed eruption was correlated with alterations in the proliferative and differentiation status of epithelial stem cells in the cervical loop area of the incisors. Similar results were obtained with in vitro studies, where inhibition of the Notch signalling by the CB103 blocker recapitulated the in vivo phenotype. In conclusion, this study demonstrates for the first time the importance of Notch2 in epithelial cell fate acquisition, dental epithelium organisation and enamel structure in rodent incisors.

## 1. Introduction

The Notch signalling pathway is an evolutionarily conserved cell communication mechanism that enables neighboring cells to acquire specific fates and further affects cell proliferation, differentiation and apoptosis within developing and regenerating tissues and organs [1,2,3]. In mammals, four Notch transmembrane receptors (Notch1, Notch2, Notch3, and Notch4) and five membrane-bound ligands (Jagged1, Jagged2, Delta-like1, Delta-like3, and Delta-like4) have been identified [2,4,5]. Ligand-receptor binding triggers successive proteolytic cleavages of the Notch receptor by Adam10 (a disintegrin and metalloprotease 10) and ɣ-secretase that enables the release of the Notch intracellular domain (NICD) and its subsequent nuclear translocation [6,7]. NICD functions as a transcriptional regulator within the nucleus by activating Notch downstream genes such as Hes1 and Hes5 [2]. Numerous studies have demonstrated that Notch pathway deletion or dysfunction severely affects neurogenesis, angiogenesis, somite formation, lymphoid, kidney and tooth development [8]. Indeed, our previous studies have shown that the Notch pathway constitutes a signalling hub throughout odontogenesis by regulating epithelial cell fates, enamel formation, and tooth morphology [9,10,11,12].

Teeth are highly mineralised organs developing through sequential and reciprocal interactions between the oral-derived dental epithelium and the neural crest-derived dental mesenchyme [9,13,14]. These continuous tissue-tissue interactions give rise to four distinct epithelial cell types, including the enamel-forming ameloblasts [15,16]. Most studies on odontogenesis have been conducted in rodent molars, due to their similarities to human molar teeth [11,17,18]. However, the rodent incisors represent another powerful biological model for studying the cellular and molecular mechanisms involved in organ development, homeostasis, and regeneration. The continuously growing rodent incisor has defined territories of stem cells, proliferating cells, differentiating cells, and mineral-secreting cells that allow a thorough assessment of tissue organisation and gene expression analysis [10,19,20,21]. Another intriguing characteristic of rodent incisors is that only their labial side is covered by enamel, since the epithelial cells of their lingual side do not differentiate into ameloblasts [22,23]. As in molars, the incisor’s labial epithelium consists of four specific cell layers (outer enamel epithelium, stratum intermedium, stellate reticulum, and inner enamel epithelium/ameloblasts), while the lingual epithelium comprises only two cell layers (outer dental epithelium and inner dental epithelium). Various epithelial stem cell populations located at the most posterior part of the labial side of the incisor, also called the labial cervical loop area, are responsible for the continuous turnover and growth of the incisor’s epithelium [20,24]. These stem cells give rise to transit-amplifying progenitors that gradually become post-mitotic and differentiate into ameloblasts. The final transition of dental epithelial stem cells to functional ameloblasts occurs through a well-orchestrated cell lineage differentiation process where positional information cues and cell polarity modifications can be assessed [25]. Previous studies have established that intercellular connection integrity within dental epithelium is crucial for proper mineral protein secretion and enamel formation [9,26,27,28,29]. Notch signalling is involved in these events, since its inhibition results in the disorganisation of incisors’ epithelium and defective enamel formation [10,26,30]. Our recent findings in incisors of *Keratin14*^Cre/+^;*Adam10^f^*^l/fl^ transgenic mice have shown that the inhibition of the Adam10/Notch signalling axis led to the loss of the stratum intermedium layer, the disorganisation of the dental epithelium and the severe malformation of the enamel [10]. Similarly, previous results on blocking Notch signalling with neutralising antibodies have demonstrated impaired interactions between ameloblasts and stratum intermedium cells, which significantly affected the structure of enamel [26]. These results establish the pivotal role of Notch signalling for dental epithelial cell fate acquisition and enamel formation. Indeed, the contribution of Notch1 in the establishment of the dental epithelium has been evaluated extensively [20]. However, there are no studies yet concerning the functional role of the Notch2 receptor during dental epithelium development and enamel formation (amelogenesis). Here, we used *Notch1*-Cre^ERT2/+^;R26^mT/mG^ and *Notch2*-Cre^ERT2/+^;R26^mT/mG^ transgenic mice and conditional *Keratin14*^Cre/+^;*Notch2*^fl/fl^ mice to study the role of Notch signalling in amelogenesis. We followed the fate of *Notch1*- and *Notch2*-expressing cells and their progenitors in incisors’ epithelium generation and demonstrated that Notch2 deletion affected dental epithelium organisation and enamel structure. Our data reveal for the first time that Notch2 is essential for maintaining dental epithelial tissue integrity and proper enamel formation.

## 2. Materials and Methods

### 2.1. Mice Handling and Ethics Statement

C57BL/6J, *Keratin14*^+/+^*;Notch2*^fl/fl^ (control), *Keratin14*^Cre/+^*;Notch2*^fl/fl^ [31], *Notch1-Cre*^ERT2/+^*;R26*^mT/mG^ and *Notch2-Cre*^ERT2*/+*^*;R26*^mT/mG^ [32] mice were used for this study. Animal housing and experimentation were performed according to the Swiss Animal Welfare Law (Act SR 455) and in compliance with the regulations of the Cantonal Veterinary Office, Zurich (License national number 32900, cantonal number 203/2020, date of approval 7 January 2022, and license national number 36516, cantonal number 195/2023, date of approval 13 March 2024). Cre-recombinase activation was induced at postnatal day 8 (PN8) by intraperitoneal administration of Tamoxifen (TAM; Sigma-Aldrich, Saint Louis, MO, USA, #T5648) to a 75 mg/kg final concentration. Postnatal pups were injected intraperitoneally first with 5-Ethynyl-2′-deoxyuridine (EdU, ABP Biosciences, Rockville, MD, USA, #ABP-A012) at PN8 to a final concentration of 50 mg/kg. At PN10, pups were injected with 5-Bromo-2′-Deoxyuridine (BrdU, Thermo Fischer Scientific, Waltham, MA, USA, #B23151) to the same final concentration, and 2 h later, they were euthanised.

### 2.2. Tissue Collection and Processing

Lower mandibles of postnatal day 10 (PN10) pups were dissected in cold 1x Phosphate-Buffered Saline, pH 7.4 (Gibco™ PBS, Thermo Fischer Scientific, MA, USA, #10-010-072) under the M80 Leica stereomicroscope (Leica Microsystems, Wetzlar, Germany). The mandibles were fixed for 48 h in 4% paraformaldehyde (PFA, Sigma-Aldrich, Saint Louis, MO, USA, #P6148) and decalcified in 0.5 M ethylenediaminetetraacetic acid (EDTA; Sigma-Aldrich, Saint Louis, MO, USA, #E9884) for 2 weeks. The mandibles were then processed for paraffin embedding or Tissue-Tek^®^ O.C.T compound (Sakura Finetek, Torrance, CA, USA, #4583) embedding for microtome or cryostat sectioning, respectively.

### 2.3. Incisor Dissection and Length Measurement

To measure the length of the incisors, lower mandibles were isolated, and incisors were dissected as previously described [33]. The obtained brightfield images were analysed, and the length of the incisors was measured in Image J (https://imagej.net/ij/index.html, accessed on 14 August 2020). At least 6 incisors from different biological samples per group were included in the measurements.

### 2.4. Histological Evaluation

Cryosections were stained using haematoxylin (Merck, Darmstadt, Germany, #109249) and eosin (Sigma-Aldrich, Saint Louis, USA, #230251-25G). Brightfield images were acquired at 20× and 40× magnification using the Leica DM750 microscope (Leica Microsystems, Wetzlar, Germany). Histological stainings were repeated more than 3 times per group and developmental stage. At least 6 samples, derived from different litters to ensure biological variability, were included in the analysis.

### 2.5. Immunostaining on Sections

Ten micrometer cryosections were used for both immunohistochemistry and immunofluorescence. Sections were permeabilised with 0.1% Triton X-100 (Sigma–Aldrich, Saint Louis, USA, #T8787) in 1x PBS for 15 min at room temperature. Antigen retrieval was performed in 10 mM citrate buffer (pH 6) at 80 °C for 30 min. Primary antibodies (all diluted 1:100) used included the following: Green fluorescent protein (GFP, Abcam, Cambridge, UK, #ab6673), Keratin14 (Biolegend, San Diego, CA, USA, #905301), Notch1 and Notch2 (Cell Signaling, Danvers, MA, USA, #5732), Claudin1 and Claudin5 (Abcam, Cambridge, UK, #ab15098, #ab15106), Claudin10 (Thermo Fischer Scientific, MA, USA, #388400), Ki67 (Abcam, Cambridge, UK, #ab16667), BrdU (Abcam, Cambridge, UK, #ab6326), Sox2 (Abcam, Cambridge, UK, #97959), Ameloblastin (Invitrogen, Waltham, MA, USA #PA5-103108), and Amelogenin (Abcam, Cambridge, UK, #ab153915). Alexa Fluor™ 488 Phalloidin (1:200 dilution; Thermo Fischer Scientific, MA, USA, #A12379) was used to stain F-actin. For immunohistochemistry, endogenous peroxidase was quenched with 1% H_2_O_2_ in methanol for 10 min. Sections were blocked with 1% Bovine Serum Albumin (BSA, Roth, Mannheim, Germany, #0163.2) and 10% Normal Goat Serum (NGS; Sigma-Aldrich, Saint Louis, USA, #NS02L), incubated with biotinylated secondary antibodies (1:250 dilution), and developed using the VECTASTAIN^®^ ABC-HRP Kit (Vector Laboratories, Newark, CA, USA, #PK-4000) and AEC Substrate (Vector Laboratories, Newark, CA, USA, #SK4200). Counterstaining was performed with toluidine blue (Sigma-Aldrich, Saint Louis, USA, #T3260), and slides were mounted in Glycergel (Agilent Dako, Santa Clara, CA, USA, #C0563). For immunofluorescence, sections were blocked with 10% donkey serum (BioWest, Nuaillé, France, #S2170-100) and 1% BSA. After overnight primary antibody incubation at 4 °C, sections were incubated with fluorophore-conjugated secondary antibodies (1:500 dilution), counterstained with 4′,6-Diamidino-2-Phenylindole (DAPI, Tocris, Bristol, UK, #5748), and mounted using a fluorescence mounting medium (Agilent Dako, Santa Clara, CA, USA, #S3023). Incorporation of EdU was detected using the Click-iT^®^ EdU Imaging Kit (Life Technologies, Carlsbad, CA, USA, #C10640). Immunostaining assays were repeated at least 3 times per group and developmental stage from different litters.

### 2.6. Scanning Electron Microscopy (SEM)

SEM evaluation was processed as previously described [10]. Briefly, 6 PN10 pups per group were euthanised, and the lower mandibles were removed and fixed with 4% PFA for 72 h at 4 °C. Then, the mandibles were dehydrated in ethanol and embedded in Technovit 7200 VLC (Heraeus Kulzer, Wehrheim, Germany). Light-polymerised blocks were mounted on aluminium stubs, polished, and coated with a 10–15 nm thick carbon layer. Mandibles were examined using a Tescan VEGA TS5316 XM SEM (Tescan, Brno, Czech Republic) operated in BSE mode. Micrographs were recorded at 20 kV and a working distance of 23 mm.

### 2.7. Micro-Computed Tomography (μCT)

mCT scans were performed using a SkyScan 1272 (Bruker BioSpin AG, Fällanden, Switzerland). All imaging parameters were identical during the examination (80 kv tube voltage, 125 μA tube current; 5 μm isotropic resolution; Al 0.5 mm filter). The reconstruction was performed using the NRecon software 2.0 (Micro Photonics, Inc., Allentown, PA, USA) with identical parameters for all samples, including histogram, beam hardening, ring artefact correction, and smoothing. A region of interest containing the incisor was generated in ImageJ 2.14.0. Enamel was individually segmented using the LABKIT plug-in of ImageJ, and the segmentation was transformed into a surface using Imaris 9.9 (Oxford Instruments, UK), from which the volume was recorded. Lower mandibles of 6 PN10 pups per group were included in the analysis.

### 2.8. Tissue Collection for Gene Expression Analysis

Upon removal of the lower mandibles, incisors were isolated as previously described [33]. The incisors were incubated with 2 U/mL of Dispase II (Sigma-Aldrich, Saint Louis, USA, #D4693) in PBS for 20 min at room temperature. The entire incisor epithelium was isolated using fine forceps and snap-frozen in liquid nitrogen. For the labial cervical loop epithelium isolation, the posterior parts of the dissected incisors were collected, and the epithelium was separated from the mesenchyme. Postnatal tissue was homogenised using a motor pestle and sonicated for 30 min using a ULTRASonik Ney-C sonicator (Blackstone-Ney, Cincinnati, OH, USA). The reverse transcription of the RNA was performed using the iScript™ cDNA Synthesis Kit (Bio-Rad, Basel, Switzerland, #1708891) following the manufacturer’s instructions. Relative mRNA expression levels were evaluated using the PowerTrack™ SYBR Green Master Mix (Thermo Fischer Scientific, MA, USA, #A46109) following the manufacturer’s instructions. The quantitative 3-step real-time polymerase chain reaction (qRT-PCR) was performed on an Illumina Eco™ (Labgene Scientific SA, Châtel-Saint-Denis, Switzerland) using 15 ng of cDNA per reaction. For each genotype and each stage, 6 independent biological replicates were analysed.

### 2.9. LS8 Dental Epithelium-like Cell Line and In Vitro Experimentation

The LS8 ameloblast-like cells, an SV40-immortalized mouse cell line, were primarily isolated from the dental epithelium of the mouse enamel organ by Dr. Malcolm L. Snead and were kindly provided by Dr. Bugueno from the University of Strasbourg [34,35,36]. LS8 cells were cultured in Dulbecco’s Modified Eagle’s Medium (DMEM; Gibco, Grand Island, NY, USA) containing 10% Foetal Bovine Serum (FBS; Gibco, Grand Island, NY, USA), 100 U/mL Penicillin, and 100 mg/mL Streptomycin at 37 °C in a 5% CO_2_ humidified atmosphere. Cells were seeded on a 12-well plate (Corning, New York, NY, USA, #353043) and treated with either 50 mM CB103 [37] or Dimethylsulfoxide (DMSO, Sigma-Aldrich, Saint Louis, USA, #D4540) (as control) for 48 h. CB103 is a selective pan-Notch pathway inhibitor that functions by blocking the interaction between the Notch intracellular domain (NICD) and the CSL (CBF1/RBPJκ, Su(H), Lag-1) transcriptional complex. CB103 acts downstream of NICD release, preventing Notch target gene activation [38]. Cell viability was assessed using the Alamar Blue assay. Specifically, DMSO- and CB103-treated cultures were incubated with Alamar blue [39] (Thermo Fischer Scientific, MA, USA, #A50100). After treatment, cells were incubated with Alamar Blue reagent (resazurin-based) according to the manufacturer’s protocol. Briefly, 10% of the total culture volume of Alamar Blue was added directly to the culture medium in each well of the plate. The cells were then incubated at 37 °C in a humidified atmosphere containing 5% CO_2_ for 1 h. Following incubation, 100 μL of supernatant from each well was carefully collected and transferred to a fresh 96-well plate. The absorbance was measured at 570 nm and 600 nm using a microplate spectrophotometer (BioTek Synergy, Agilent Technologies, Santa Clara, CA, USA). The degree of Alamar Blue reduction, which correlates with cell viability, was calculated by comparing the absorbance values at the two wavelengths according to the manufacturer’s formula. Control wells containing untreated viable cells were used to represent 100% viability, and blank wells containing only medium and reagents were used to subtract background. All measurements were performed in triplicate, and the results were expressed as a percentage of viable cells relative to the untreated control.

For immunofluorescence studies, cultures treated with DMSO or CB103 were fixed with 4% PFA overnight, washed with PBS several times, permeabilised with 0.1% Triton X-100, and finally incubated with blocking buffer consisting of 10% donkey serum and 1% BSA for 30 min. Immunofluorescence staining proceeded as described above. For RNA isolation, 1 mL of TRIzol reagent (Thermo Fischer Scientific, MA, USA, #15596026) was added to each well, and then the cultures were scraped manually using a 240 mm cell scraper (TPP Techno Plastic Products AG, Trasadingen, Switzerland, #20220351). The cell medium was collected and homogenised using a motor pestle and sonicated for 30 min using a ULTRASonik Ney-C sonicator (Blackstone-Ney, Cincinnati, OH, USA). The reverse transcription and qRT-PCR were performed as described above, and all the measurements were performed in triplicate.

### 2.10. Quantification and Statistical Analysis

Quantified data were analysed by Student’s *t*-test using Microsoft Excel software. The value *p* < 0.05 was considered statistically significant. Statistical significance was assessed as follows: ^ns^, *p* > 0.05; *, 0.01 < *p* ≤ 0.05; **, 0.001 < *p* ≤ 0.01; ***, 0.0001 < *p* ≤ 0.001; ****, *p* ≤ 0.0001.

## 3. Results

### 3.1. Distribution of the Notch1 and Notch2 Proteins in Prenatal and Early Postnatal Incisors

**Figure 1:**
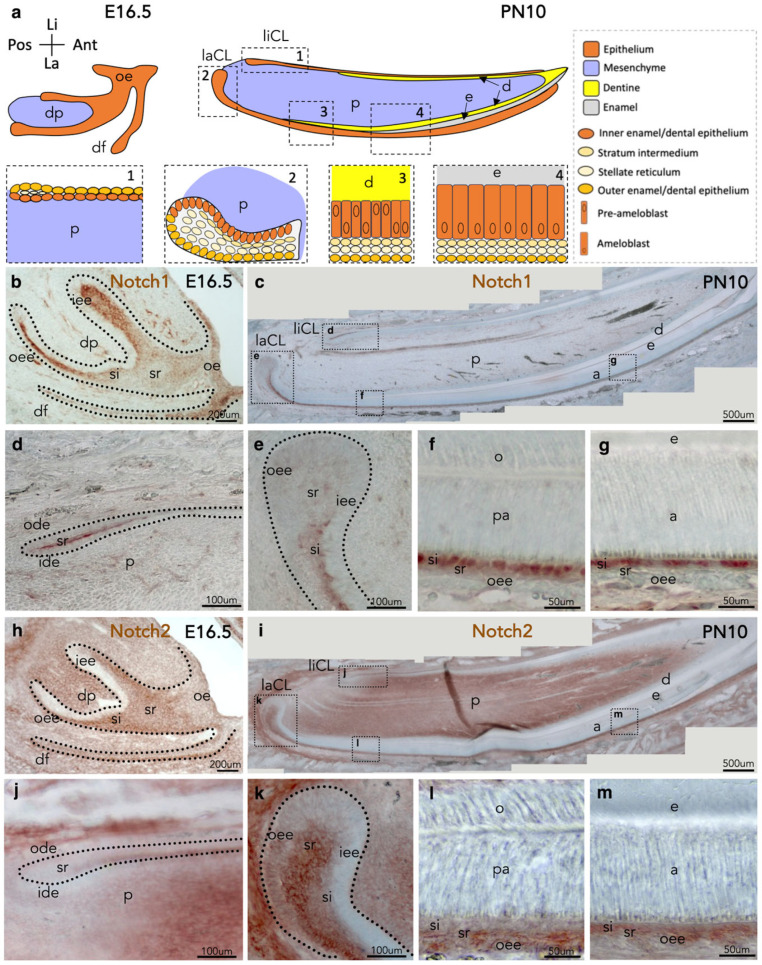
Immunohistochemical localisation of the Notch1 and Notch2 proteins in prenatal and early postnatal incisors. (**a**) Schematic representation of an embryonic day 16.5 (E16.5) and a postnatal day 10 (PN10) incisor. Dashed boxes indicate high-magnifications of 4 different areas. (**b**) Notch1 protein distribution in a developing incisor at E16.5. Dotted lines indicate the borders of the incisor’s epithelium. (**c**) Notch1 localisation in a PN10 incisor. Dashed boxes indicate high-magnifications of the lingual cervical loop (**d**), the labial cervical loop (**e**), the pre-ameloblast (**f**), and the ameloblast (**g**) compartments of the incisor. Dotted lines in (**d**,**e**) indicate the borders of the incisor epithelium. (**h**) Distribution of the Notch2 protein in an E16.5 incisor. Dotted lines indicate the epithelial borders. (**i**) Notch2 protein distribution in a PN10 incisor. Dotted boxes designate high-magnifications of the lingual cervical loop (**j**), the labial cervical loop (**k**), the pre-ameloblast (**l**), and ameloblast (**m**) areas of the incisor. Dotted lines in (**j**,**k**) indicate the borders of the incisor epithelium. Immunostaining for Notch1 and Notch2 has been repeated more than 3 times per developmental stage. Abbreviations: a, ameloblasts; d, dentine; dp, dental papilla; e, enamel; ide, inner dental epithelium; iee, inner enamel epithelium; laCL, labial cervical loop; liCL, lingual cervical loop; o, odontoblasts; oe, oral epithelium; ode, outer dental epithelium; oee, outer enamel epithelium; p, dental pulp; pa, pre-ameloblasts; si, stratum intermedium; sr, stellate reticulum.

We first analysed the distribution of the Notch1 and Notch2 proteins in developing incisors during prenatal and early postnatal stages (Figure 1a) by immunohistochemistry in wild-type mice. Notch1 and Notch2 are expressed in the oral epithelium, the dental furrow, the lingual and labial epithelia, and the dental papilla of the incisor at embryonic day 16.5 (E16.5) (Figure 1b,h). More specifically, Notch1 protein was strongly expressed in cells of the stratum intermedium (si) and stellate reticulum (sr) but absent from the outer enamel epithelium (oee) and the inner enamel epithelium (iee) layers (Figure 1b). Strong Notch2 staining was localised in the sr and oee, while it was slightly detected in si cells and absent from the iee layer (Figure 1h). In the dental papilla, Notch1 protein was localised in the vessels, while Notch2 was detected in most mesenchymal cells.

At postnatal day 10 (PN10), Notch1 protein was detected predominantly in the si layer (Figure 1c) and sparsely in sr cells of the labial and lingual cervical loops (Figure 1d,e) and pre-ameloblasts of the labial epithelium (Figure 1f). This expression pattern was progressively restricted to si cells during ameloblast differentiation (Figure 1g). At this stage, Notch2 protein was principally detected in cells of the oee and sr layers and sporadically in si cells of the labial epithelium of the incisors (Figure 1i–m). With the onset of iee cell differentiation into ameloblasts, Notch2 staining became more pronounced in the si cells (Figure 1l,m). Notch2 protein was not detected in the lingual cervical loop (Figure 1j). Like the expression pattern observed at prenatal stages, Notch1 and Notch2 proteins were absent from ameloblasts of the labial epithelium. Strong Notch2 immunostaining was detected in the dental pulp and periodontal mesenchymal tissues (Figure 1c,i).

### 3.2. Lineage Tracing of Notch1-Expressing Cells and Their Progeny in the Epithelium of Early Postnatal Incisors

**Figure 2:**
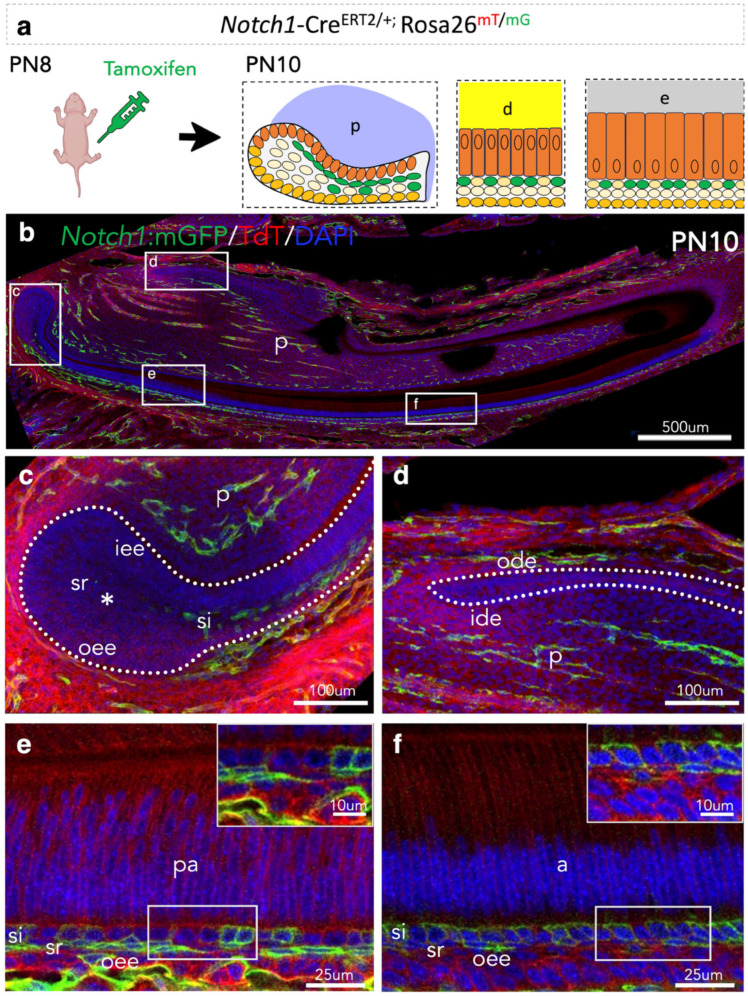
Lineage tracing of *Notch1*-expressing cells and their progeny in early postnatal incisors. (**a**) Schematic representation of the experimental setup and distribution of GFP-expressing cells in the labial cervical loop, and the pre-ameloblast and ameloblast compartments of the PN10 incisor. (**b**) Overview of the immunofluorescent staining against Notch1/GFP and TdTomato in PN10 *Notch1*-Cre^ERT2^;R26^mT/mG^ incisors. Dashed boxes indicate high-magnifications of the labial cervical loop (**c**), the lingual cervical loop (**d**), and the pre-ameloblast (**e**) and ameloblast (**f**) compartments of the incisor. Dotted lines in (**c**,**d**) indicate the borders of the incisor epithelium. Dotted boxes (lower part) in (**e**,**f**) show areas that are magnified in the inserts (upper part). The asterisk in (**c**) indicates the absence of GFP in this part of the labial cervical loop. Nuclei are stained with DAPI in blue. Abbreviations: a, ameloblasts; d, dentine; e, enamel; ide, inner dental epithelium; iee, inner enamel epithelium; ode, outer dental epithelium; oee, outer enamel epithelium; p, pulp; pa, pre-ameloblasts; si, stratum intermedium; sr, stellate reticulum.

We then performed lineage tracing experiments using early postnatal *Notch1*-Cre^ERT2/+^;R26^mT/mG^ transgenic mice to monitor the involvement of *Notch1*-expressing cells and their progeny in establishing incisors’ epithelium. Green fluorescent protein (GFP) is activated in *Notch1*-expressing cells upon tamoxifen administration, and, therefore, we can follow their fates within the dental epithelium of early postnatal incisors (Figure 2a).

We performed a short lineage tracing by injecting PN8 pups with tamoxifen and euthanised them at PN10 for collecting the incisors. This approach allowed us to assess the spatial distribution of cells with recent Notch1 expression, providing insight into its transient activity during early postnatal development. *Notch1*-positive cells were detected in the epithelium and dental pulp of the PN10 incisors (Figure 2b). In the labial cervical loop epithelium, *Notch1*-positive cells were mostly observed in the si layer and sparsely in the sr layer (Figure 2c), but were absent from its most posterior part (Figure 2c). At the transition and pre-ameloblast/ameloblast compartments of the incisor, *Notch1*-positive cells were sparsely detected in the si, sr, and oee layers (Figure 2e,f). Interestingly, while immunohistochemistry at PN10 showed no detectable Notch1 protein in the oee (Figure 1f), lineage tracing revealed *Notch1*-positive cells in this layer (Figure 2e). This discrepancy likely reflects a temporal difference between transient Cre activity driven by the Notch1 promoter and the later downregulation of endogenous Notch1 protein expression. *Notch1*-positive cells were absent from the lingual epithelium of the incisor (Figure 2d).

### 3.3. Lineage Tracing of Notch2-Expressing Cells and Their Progeny in the Epithelium of Early Postnatal Incisors

**Figure 3:**
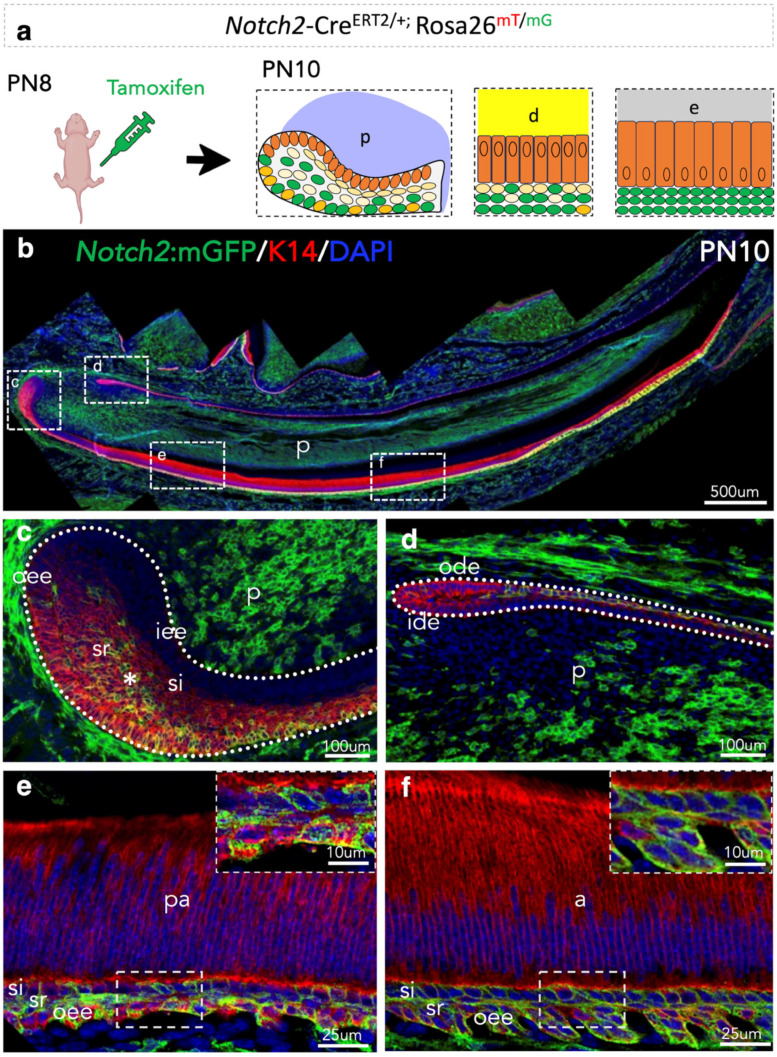
Lineage tracing of *Notch2*-expressing cells and their progeny in early postnatal incisors. (**a**) Schematic representation of the experimental setup and distribution of *Notch2*-expressing cells and their progeny in PN10 incisors. (**b**) Overview of the immunofluorescent staining against Notch2-driven GFP and protein Keratin14 in PN10 *Notch2*-Cre^ERT2^;R26^mT/mG^ incisors. Dashed boxes indicate high-magnifications of the labial cervical loop (**c**), the lingual cervical loop (**d**), and the pre-ameloblast (**e**) and ameloblast (**f**) compartments of the incisor. Dotted lines in (**c**,**d**) indicate the borders of the incisor epithelium. Dotted boxes (lower part) in (**e**,**f**) show areas that are magnified in the inserts (upper part). The asterisk in (**c**) indicates the presence of GFP in this part of the labial cervical loop. Nuclei are stained with DAPI in blue. Abbreviations: a, ameloblasts; d, dentine; e, enamel; ide, inner dental epithelium; iee, inner enamel epithelium; ode, outer dental epithelium; oee, outer enamel epithelium; p, pulp; pa, pre-ameloblasts; si, stratum intermedium; sr, stellate reticulum.

Next, we explored the contribution of *Notch2*-expressing cells and their progeny in the establishment of incisors’ epithelium by performing lineage tracing in *Notch2*-Cre^ERT2/+^;R26^mT/mG^ transgenic mice upon tamoxifen administration (Figure 3a). *Notch2*-expressing cells and their progeny were detected in the epithelium and dental pulp of the PN10 incisor (Figure 3b). Extensive Notch2-driven GFP labelling was observed at the anterior, but not posterior, labial part of the labial cervical loop epithelium of the incisor (Figure 3c). *Notch2*-positive cells were sporadically found in the outer dental epithelium (ode) of the lingual part of the PN10 incisors (Figure 3d). Few *Notch2-*expressing cells were observed in the si layer of the pre-ameloblast compartment of the incisor (Figure 3e), while all cells of the si layer of the ameloblast compartment were *Notch2*-positive (Figure 3f).

### 3.4. Epithelial Notch2 Deletion in Incisors Leads to Shorter Incisors and Enamel Malformation

**Figure 4:**
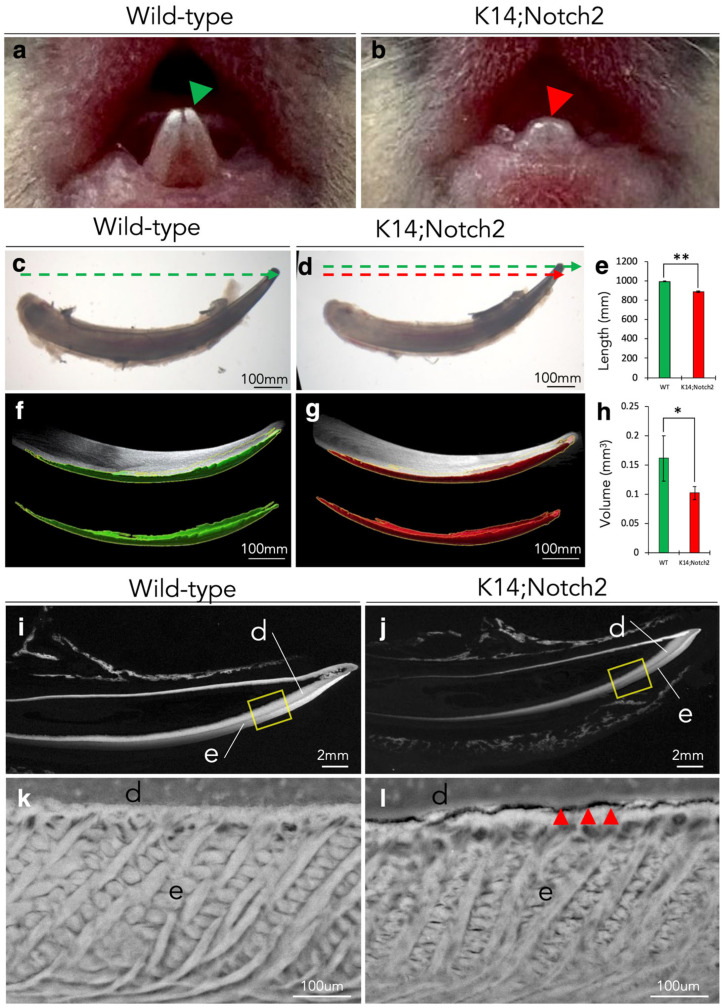
Epithelial Notch2 deletion results in shorter incisors with enamel defects. Representative macroscopic images of mandibular incisors in PN10 wild-type (**a**) and (*Keratin14*^Cre/+^;*Notch2*^fl/fl^) K14;Notch2 knock-out (**b**) mice. Green and red arrowheads highlight the tips of the erupted incisors in wild-type and Notch2 mutant mice, respectively. Brightfield images of isolated PN10 wild-type (**c**) and K14;Notch2 knock-out (**d**) incisors. The green arrow in (**c**) shows the length of the control incisor, and the red arrow in (**d**) indicates the length of the mutant incisor. Quantifications of the incisors’ length (**e**) in wild-type and Notch2 mutant mice. Surface segmentation from mCT imaging of entire incisors and their enamel compartment from wild-type (**f**) and mutant (**g**) mice. Green and red indicate the enamel compartments in wild-type and Notch2 mutant incisors, respectively. Quantifications of the incisors’ total enamel volume (**h**) in wild-type and Notch2 mutant mice. The graphs represent the mean and standard deviation (n = 6). Statistical significance was calculated with Student *t*-test. *, *p* ≤ 0.05; **, *p* ≤ 0.01. SEM images of PN10 wild-type (**i**,**k**) and Notch2 mutant (**i**,**j**) incisors. Yellow boxes in (**i**,**j**) indicate magnified areas showing the enamel structure in (**k**,**l**), respectively. Red arrowheads in (**l**) show a defect in the dentine-enamel junction of a mutant incisor. Abbreviations: d, dentine; e, enamel.

To investigate the role of the Notch2 receptor in amelogenesis, we selectively knocked out the *Notch2* gene in the incisor’s epithelium using the *Keratin14*^Cre/+^;*Notch2*^fl/fl^ transgenic mouse line. At PN10, the incisors of wild-type pups have already started to erupt, and their tips have already appeared well in the oral cavity (Figure 4a, green arrowhead). However, the eruption of Notch2 mutant incisors was significantly delayed, with their tips starting to appear (Figure 4b, red arrowhead). We then extracted the incisors from the mandibles and measured the length of the isolated wild-type and Notch2 mutant incisors. The comparison between wild-type (Figure 4c, green arrow) and mutant incisors (Figure 4d, red arrow) revealed that Notch2 deletion leads to shorter than normal incisors (Figure 4c–e).

For further morphological analysis, wild-type and Notch2 mutant incisors were analysed using microcomputed tomography (mCT) and scanning electron microscopy (SEM). mCT analysis and enamel segmentation revealed that the total volume of enamel in mutant incisors was significantly decreased compared to wild-type incisors (Figure 4f–h). SEM analysis indicated that the enamel rods in the enamel of mutant incisors were misaligned and improperly arranged compared to the enamel of the wild-type incisors, leading to a highly disorganised enamel structure (Figure 4i–l). Cracks in the dentine-enamel interface were also revealed upon SEM analysis in mutant incisors (Figure 4l).

### 3.5. Loss of Notch2 Results in Defective Dental Epithelium

**Figure 5:**
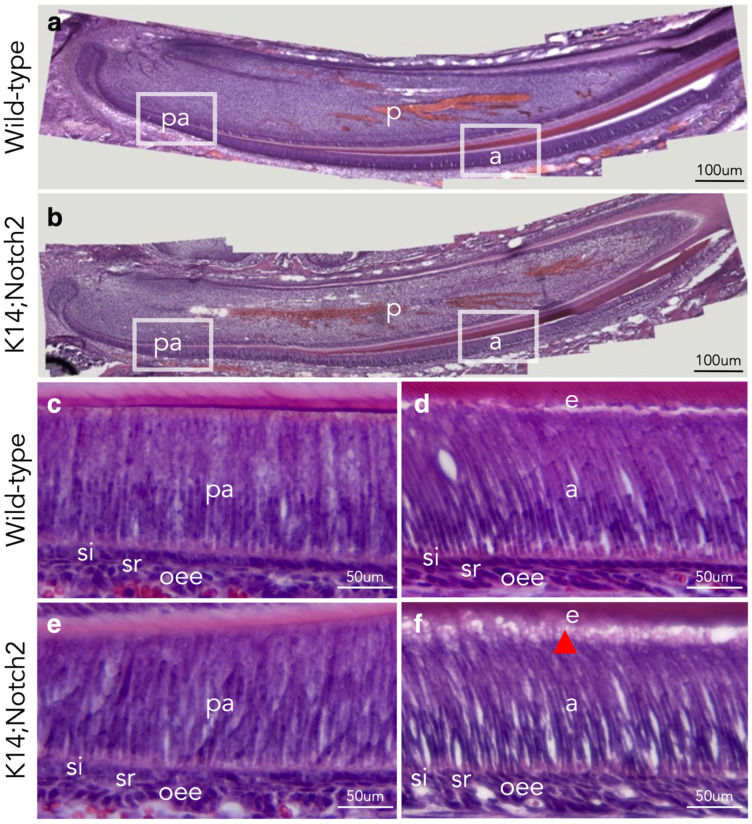
Epithelial Notch2 deletion leads to disorganisation of the dental epithelium. Haematoxylin and eosin staining on longitudinal sections of wild-type and (*Keratin14*^Cre/+^;*Notch2*^fl/fl^) K14;Notch2 knock-out PN10 incisors. Overview of stained wild-type (**a**) and mutant (**b**) incisors. White boxes in (**a**,**b**) indicate magnified areas shown in (**c**–**f**). High magnifications showing the pre-ameloblasts (**c**,**e**) and ameloblasts (**d**,**f**) in incisors of wild-type (**c**,**d**) and mutant mice (**e**,**f**). The red arrow in (**f**) indicates the detachment of the ameloblasts from the enamel. Abbreviations: a, ameloblasts; e, enamel; iee, inner enamel epithelium; o, odontoblasts; oee, outer enamel epithelium; p, pulp; pa, pre-ameloblasts; si, stratum intermedium; sr, stellate reticulum.

We then performed histological analysis in PN10 wild-type and *Keratin14*^Cre/+^;*Notch2*^fl/fl^ knock-out incisors to investigate the cellular mechanisms underlying the enamel phenotype in mutant mice (Figure 5a,b). Haematoxylin and eosin histological staining indicated that the pre-ameloblast/ameloblast layer of mutant incisors was disordered and depolarised, as opposed to the highly organised layer of wild-type incisors (Figure 5c–f). Histology also revealed defects in the connection between the ameloblasts and enamel layer of mutant incisors (Figure 5f). Furthermore, cells of the sr and oee layers were misaligned and/or intermingling in the incisors of *Keratin14*^Cre/+^;*Notch2*^fl/fl^ mice.

### 3.6. Aberrant Enamel Protein Distribution and Enamel Gene Expression in Dental Epithelium upon Notch2 Deletion

**Figure 6:**
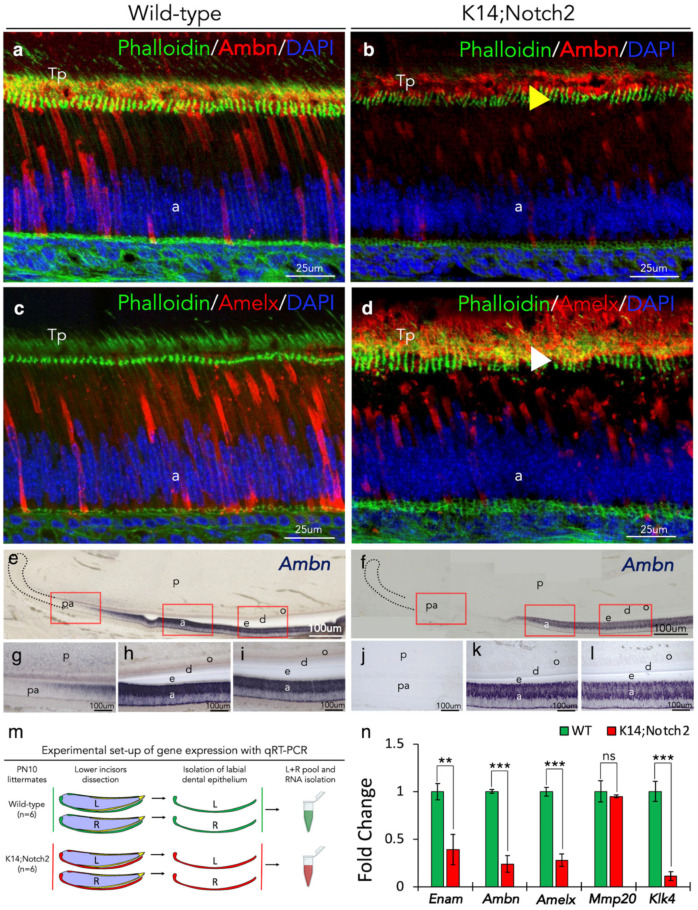
Aberrant expression of enamel-specific proteins and genes in incisors’ epithelium upon Notch2 deletion. (**a**–**d**) Immunofluorescent staining (red colour) against Ameloblastin (**a**,**b**) and Amelogenin (**c**,**d**) proteins in incisors of wild-type (**a**,**c**) and (*Keratin14*^Cre/+^;*Notch2*^fl/fl^) K14;Notch2 knock-out (**b**,**d**) PN10 mice. Phalloidin immunofluorescence (green colour) indicates the actin filaments of the cytoskeleton (**a**–**d**). Yellow (**b**) and white (**d**) arrowheads show the accumulation of Ameloblastin (**b**) and Amelogenin (**c**) staining in K14;Notch2 knock-out incisors. DAPI blue staining indicates the cell nuclei. Immunostaining was performed more than 3 times. (**e**–**l**) In situ hybridisation for *Ameloblastin* (*Ambn*) on longitudinal sections of incisors from wild-type (**e**) and K14;Notch2 knock-out (**f**) PN10 mice. Red boxes in (**e**,**f**) highlight magnified areas of pre-ameloblasts (**g**,**j**) and ameloblasts (**h**,**i**,**k**,**l**) in wild-type (**e**,**g**–**i**) and K14;Notch2 knock-out (**f**,**j**–**l**) incisors. Note the delay of *Ambn* expression in mutant incisors compared to wild-type incisors by comparing (**g**–**j**). In situ hybridisation studies have been performed more than 3 times. (**m**) Schematic representation of the isolation of the epithelium from PN10 wild-type and K14;Notch2 knock-out incisors for the realisation of RT qPCR analysis. (**n**) Quantitative mRNA analysis of genes involved in the process of amelogenesis shows downregulation of most genes, such as *Enamelin* (*Enam*), *Ambn*, *Amelogenin* (*Amelx*), *Metalloproteinase-20 (Mmp20*) and *Kallikrein-4* (*Klk4*). The graphs represent the mean and standard deviation (n = 6). Statistical significance was calculated with Student *t*-test. **, *p* ≤ 0.01; ***, *p* ≤ 0.001; ^ns^, *p* > 0.05. Abbreviations: a, ameloblasts; d, dentine; e, enamel; o, odontoblasts; p, dental pulp; pa, pre-ameloblasts; Tp, Tomes’ processes.

To explore the potential effects of epithelial disorganisation in amelogenesis, we performed co-staining for Phalloidin/Ameloblastin and Phalloidin/Amelogenin. Immunostaining for the enamel-specific proteins Ameloblastin and Amelogenin revealed their disordered distribution and accumulation in the apical end of the ameloblasts of *Keratin14*^Cre/+^;*Notch2*^fl/fl^ incisors. Furthermore, the phalloidin-mediated actin staining confirmed the disorientation of ameloblast processes and total disorganisation of the epithelium of mutant incisors (Figure 6a–d). To further explore if these changes in the Notch2 knock-out epithelium also affected the timing of ameloblast differentiation, we performed in situ hybridisation for *Ameloblastin* (*Ambn)*, one of the early genes of ameloblast differentiation. We showed that *Ambn* expression was significantly delayed in mutant incisors compared to wild-type incisors (Figure 6e–l). Thereafter, we investigated the expression of important genes for amelogenesis. Quantitative reverse transcription PCR (qRT-PCR) analysis in epithelial cells from wild-type and mutant incisors demonstrated significant downregulation in the expression of these genes, including *Enamelin* (*Enam*), *Ambn*, *Amelogenin* (*Amelx*), and *Kallikrein-4 (Klk4*) (Figure 6m,n).

### 3.7. Cell-to-Cell Adhesion Disruption in Incisors’ Epithelium upon Notch2 Deletion

**Figure 7:**
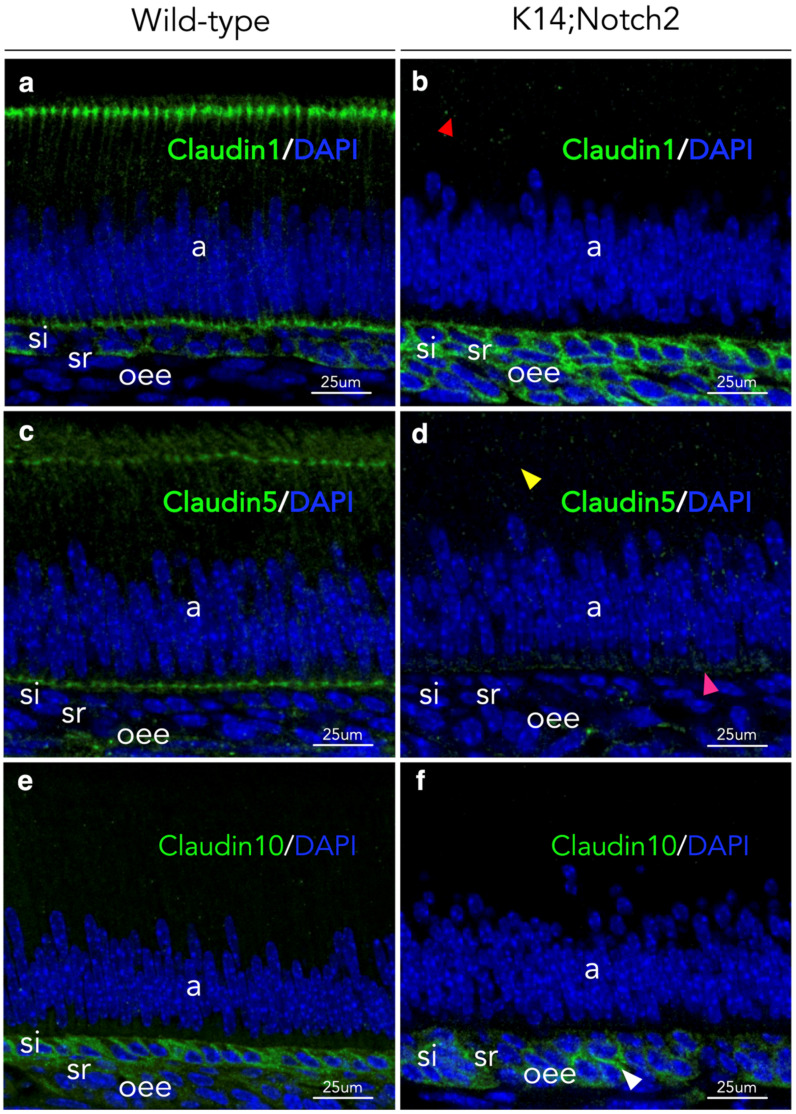
Notch2 deletion affects cell-to-cell adhesion in incisors’ epithelium. Immunofluorescent staining against the tight junction proteins Claudin1 (**a**,**b**), Claudin5 (**c**,**d**) and Claudin10 (**e**,**f**) (green colour) in the epithelium of PN10 wild-type (**a**,**c**,**e**) and mutant (**b**,**d**,**f**) incisors. DAPI staining in blue shows the cell nuclei. The red arrowhead in (**b**) shows the absence of Claudin1 staining in the apical part of the ameloblasts in mutant incisors. The yellow and magenta arrowheads in (**d**) indicate the lack of Claudin5 staining in the apical and basal parts of the ameloblasts of Notch2 knock-out incisors. The white arrowhead in (**f**) indicates the abnormal distribution of Claudin10 in the epithelium of mutant incisors. Immunostaining has been repeated more than 3 times. Abbreviations: a, ameloblasts; oee, outer enamel epithelium; sr, stellate reticulum; si, stratum intermedium.

To further evaluate the epithelial disorganisation in *Keratin14*^Cre/+^;*Notch2*^fl/fl^ incisors, we performed immunostaining to detect the tight junction-specific proteins Claudins and to analyse their localisation. The staining revealed dramatic changes in their distribution and expression pattern in the ameloblasts and the rest of the mutant incisors’ epithelium (Figure 7). More precisely, Claudin1 staining was detected in the apical and basal parts of the ameloblasts and si cells of wild-type incisors (Figure 7a). In mutant incisors, the Claudin labelling was absent in the ameloblasts, but strong staining was observed in all other epithelial cells (Figure 7b). Claudin5 labelling was detected in the apical and basal parts of the ameloblasts of wild-type incisors, but the signal was very faint or undetectable in the ameloblasts of *Keratin14*^Cre/+^;*Notch2*^fl/fl^ incisors (Figure 7c,d). Claudin10 staining was absent from the ameloblasts, intense in si cells, and weak in sr and oee cells of wild-type incisors (Figure 7e). In the incisors of *Keratin14*^Cre/+^;*Notch2*^fl/fl^ mice, irregular weak labelling was detected in si, sr and oee cells (Figure 7f).

### 3.8. Modifications in the Epithelial Stem Cell Niche of Incisors upon Notch2 Deletion

**Figure 8:**
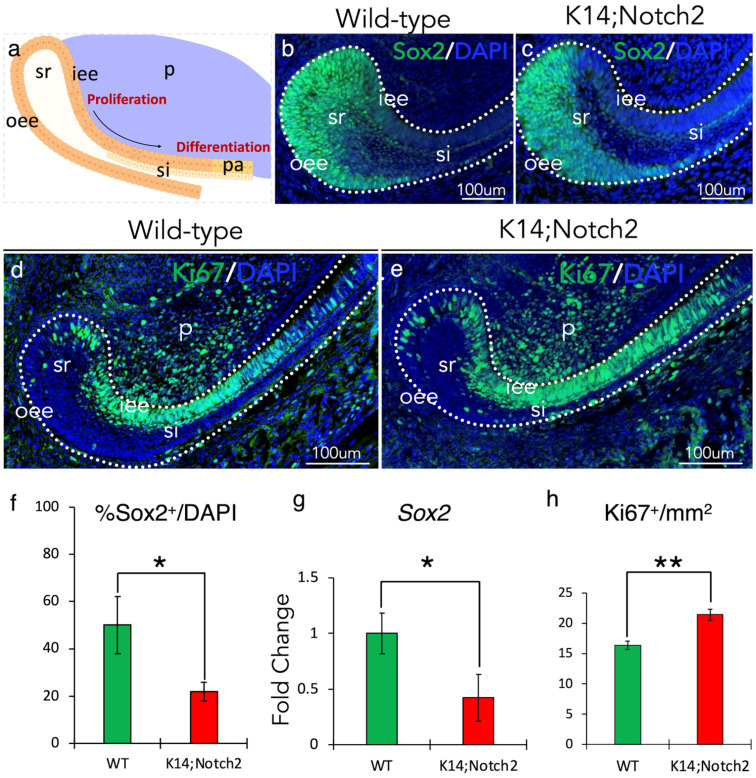
Notch2 deletion results in modifications in incisors’ epithelial stem cell niche. (**a**) Schematic representation of the labial cervical loop, where epithelial stem cells divide slowly and give rise to progenitor cells. (**b**,**c**) Immunofluorescent staining against the Sox2 protein (green colour) marking epithelial stem cells in the cervical loop of wild-type (**b**) and mutant (**c**) incisors. Cell nuclei in blue are stained with DAPI. (**d**,**e**) Immunofluorescent staining against the cell proliferation marker Ki67 (green colour) in the posterior part of wild-type (**d**) and (*Keratin14*^Cre/+^;*Notch2*^fl/fl^) K14;Notch2 knock-out (**e**) incisors. Cell nuclei in blue are stained with DAPI. (**f**) Quantification of Sox2-positive (Sox2^+^) cells in the cervical loop of wild-type and K14;Notch2 knock-out incisors. (**g**) Quantification (qPCR analysis) of *Sox2-*expressing cells in the cervical loop of wild-type and mutant incisors. (**h**) Quantification of Ki67-positive (Ki67^+^) cells in the posterior part of wild-type and mutant incisors. Cell nuclei in blue are stained with DAPI. Graphs show mean and standard deviation. For all quantifications, n = 6. Statistical significance was calculated with Student *t*-test. *, *p* ≤ 0.05; **, *p* ≤ 0.01. Abbreviations: iee, inner enamel epithelium; oee, outer enamel epithelium; p, dental pulp; si, stratum intermedium; sr, stellate reticulum.

We then investigated whether epithelial Notch2 deletion disturbs the physiology of the epithelial stem cell niche, thus contributing to the delayed eruption and smaller size of mutant incisors. Stem cells at the labial cervical loop are the main source of all dental epithelial cell populations, including pre-ameloblasts/ameloblasts, and ensure the continuous epithelial turnover and growth of the incisors [20,24] (Figure 8a).

To explore whether epithelial Notch2 deletion could affect the number of stem cells in incisors, we performed immunostaining against the dental epithelial stem cell marker Sox2. Strong nuclear Sox2 immunofluorescence labelling was detected in stem cells at the cervical loop of both wild-type (Figure 8b) and *Keratin14*^Cre/+^;*Notch2*^fl/fl^ incisors (Figure 8c). A reduction of Sox2-positive cells in mutant incisors was observed compared to wild-type ones (Figure 8f). qRT-PCR analyses in isolated epithelial cervical loops from wild-type and mutant incisors confirmed these results, showing significant *Sox2* mRNA downregulation in mutant incisors (Figure 8g).

To investigate whether the reduction of Sox2-positive cells in mutant incisors led to a modification in the number of progenitors, we performed immunostaining against the cell proliferation marker Ki67. Immunofluorescent nuclear labelling was observed in epithelial stem cells at the cervical loop, in transient amplified progenitor cells (iee and si), and mesenchymal dental pulp cells of both wild-type (Figure 8d) and mutant incisors (Figure 8e). The number of Ki67-positive progenitor cells was significantly increased in the epithelium of mutant incisors when compared to wild-type incisors (Figure 8h).

### 3.9. Notch2 Deletion Affects the Notch Signalling Pathway in the Incisors’ Epithelium

**Figure 9:**
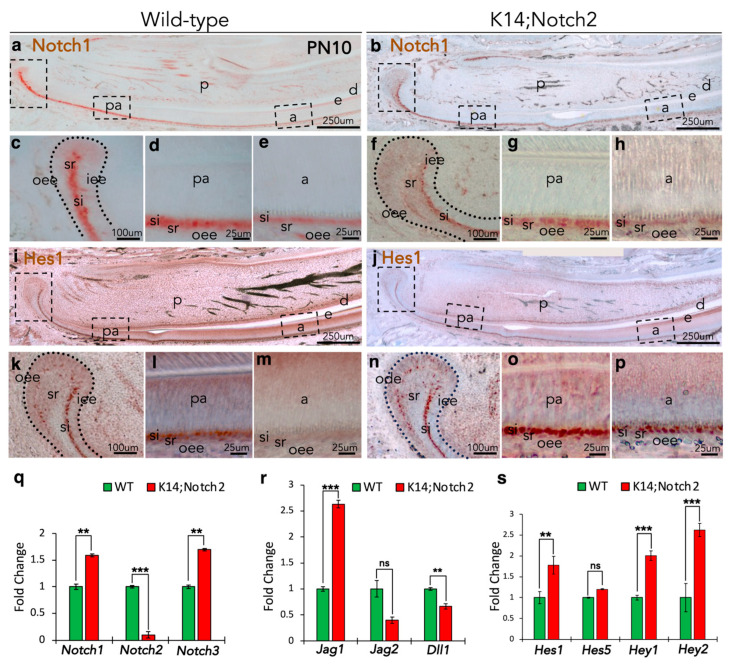
Notch2 epithelial deletion leads to Notch signalling alterations in early postnatal incisors. (**a**,**b**) Overview of Notch1 immunostaining in longitudinal sections of PN10 wild-type (**a**) and (*Keratin14*^Cre/+^;*Notch2*^fl/fl^) K14;Notch2 knock-out (**b**) incisors. Dashed boxes in (**a**,**b**) show magnified areas in (**c**–**e**) and (**f**–**h**), respectively. (**c**–**e**) Higher magnifications of the cervical loop (**c**), pre-ameloblast (**d**) and ameloblast (**e**) compartments of wild-type incisors. (**c**–**e**) Higher magnifications of the cervical loop (**f**), pre-ameloblast (**g**) and ameloblast (**h**) compartments of mutant incisors. (**i**,**j**) Overview of Hes1 immunostaining in longitudinal sections of PN10 wild-type (**i**) and mutant (**j**) incisors. Dashed boxes in (**i**,**j**) show magnified areas in (**k**–**m**) and (**n**–**p**), respectively. (**k**–**m**) Higher magnifications of the cervical loop (**k**), pre-ameloblast (**l**) and ameloblast (**m**) compartments of wild-type incisors. (**n**–**p**) Higher magnifications of the cervical loop (**n**), pre-ameloblast (**o**) and ameloblast (**p**) compartments of mutant incisors. Immunostainings for Notch1 and Hes1 have been repeated more than 3 times. (**q**–**s**) Quantification of *Notch1*, *Notch2*, *Notch3* (**q**), *Jagged1* (*Jag1*), *Jagged2* (*Jag2*), *Delta-like1* (*Dll1*) (**r**), *Hes1*, *Hes5*, *Hey1*, and *Hey2* expression in the epithelium of PN10 wild-type and mutant incisors. Graphs show mean and standard deviation. For all quantifications, n = 6. Statistical significance was calculated with Student *t*-test. **, *p* ≤ 0.01; ***, *p* ≤ 0.001; ^ns^, *p* > 0.05. Abbreviations: a, ameloblasts; d, dentine; e, enamel; iee, inner enamel epithelium; o, odontoblasts; oee, outer enamel epithelium; p, dental pulp; pa, pre-ameloblasts; si, stratum intermedium; sr, stellate reticulum.

To identify whether Notch2 loss affects the expression of other molecules involved in the Notch pathway, we performed immunostaining and qRT-PCR analyses in PN10 wild-type and mutant incisors. We first analysed by immunohistochemistry the distribution of Notch1 protein in wild-type (Figure 9a) and *Keratin14*^Cre/+^;*Notch2*^fl/fl^ (Figure 9b) incisors. Our results show that Notch1 labelling was restricted to cells of the si layer and a part of the cervical loop of wild-type incisors (Figure 9a,c–e). In mutant incisors, Notch1 staining was also localised in the si of the cervical loop (Figure 9b,f–h) and the pre-ameloblast compartment of the incisor (Figure 9g).

To further explore Notch signalling activation, we performed immunostaining against the Notch target molecule Hes1 (Figure 9i,j). Our results show that, similarly to Notch1, Hes1 labelling was restricted to cells of the si layer and a part of the cervical loop of wild-type incisors (Figure 9i,k–m). In mutant incisors, Hes1 staining was also localised in the si and cervical loop (Figure 9j,n–p), but strong staining was also observed in cells of the si layer of the ameloblast compartment of the incisor (Figure 9p).

Thereafter, we investigated the expression of several other genes of the Notch pathway. qRT-PCR analysis in epithelial cells from wild-type and mutant incisors demonstrated significant dysregulation in the expression of these genes. The expression of *Notch1* and *Notch3* genes was upregulated in the epithelium of mutant incisors, while *Notch2* expression was significantly downregulated, thus confirming the epithelial Notch2 deletion (Figure 9q). Interestingly, *Jagged1* and *Dll1* expression were significantly increased in the epithelium of mutant mice compared to wild-type ones, whereas *Jagged2* expression did not vary between mutant and wild-type incisors (Figure 9r). Finally, the expression of the Notch target genes *Hes1*, *Hes5*, *Hey1* and *Hey2* was significantly upregulated in the epithelium of mutant incisors compared to wild-type incisors (Figure 9s).

### 3.10. Notch Signalling Pharmacological Inhibition In Vitro Recapitulates the In Vivo Keratin14^Cre/+^;Notch2^fl/fl^ Incisors’ Phenotype

**Figure 10:**
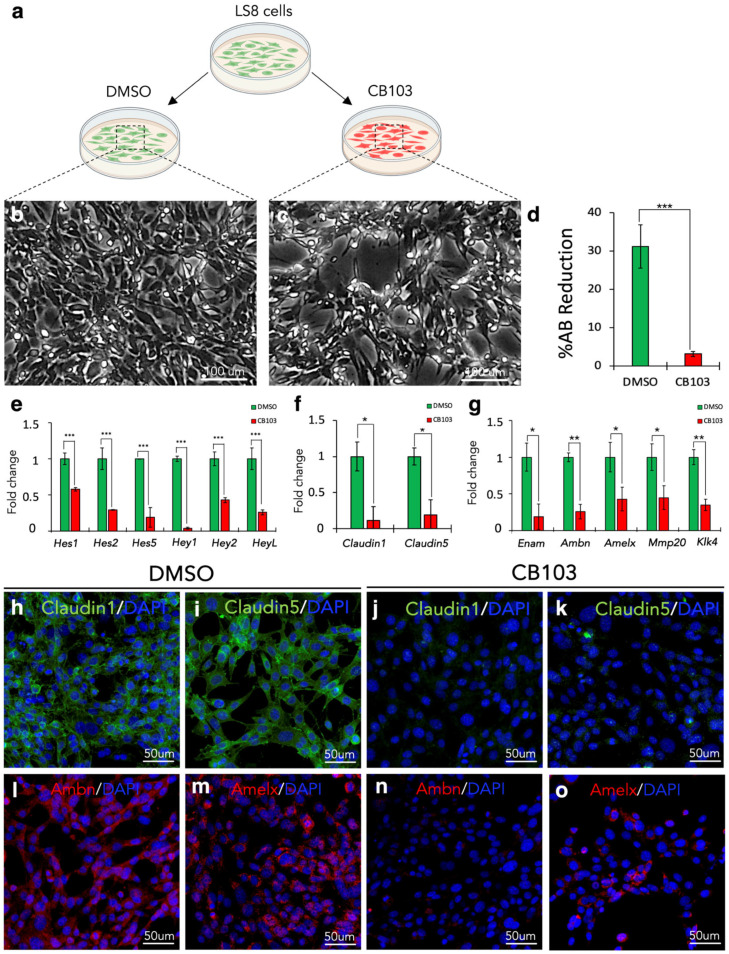
Pharmacological Notch signalling inhibition in vitro recapitulates the *Keratin14*^Cre/+^;*Notch2*^fl/fl^ incisors’ phenotype. (**a**) Schematic representation of LS8 dental epithelial cells cultured in vitro in the presence of DMSO (control) and the Notch inhibitor CB103. (**b**,**c**) Brightfield images show the phenotype of LS8 cells cultured for 2 days in the presence of DMSO (**b**) and CB103 (**c**). (**d**) Quantification of the percentage of Alamar Blue in LS8 cells cultured for 2 days in the presence of either DMSO or CB103. (**e**,**g**) mRNA quantification of the Notch effector genes *Hes1*, *Hes2*, *Hes5*, *Hey1*, *Hey2* and *HeyL* (**e**), the tight junction genes *Claudin1* and *Claudin5* (**f**) and amelogenesis-related genes *Enam*, *Ambn*, *Amelx*, *Mmp20* and *Klk4* (**g**) in LS8 cells cultured for two days in the presence of either DMSO (control) or CB103. All graphs show mean and standard deviation (n = 6). Statistical significance was calculated with Student’s *t*-test. *, *p* ≤ 0.05; **, *p* ≤ 0.01; ***, *p* ≤ 0.001. (**h**–**k**) Immunofluorescent staining (green colour) against the tight junction proteins Claudin1 (**h**,**j**) and Claudin5 (**i**,**k**) in LS8 cells cultured for two days in the presence of either DMSO (**h**,**i**) or CB103 (**j**,**k**). Cell nuclei are stained with DAPI (blue colour). (**l**–**o**) Immunofluorescent staining (red colour) against the Ameloblastin (**l**,**n**) and Amelogenin (**m**,**o**) proteins in LS8 cells cultured for two days in the presence of either DMSO (**l**,**m**) or CB103 (**n**,**o**). Cell nuclei are stained with DAPI (blue colour).

The changes observed in the expression of all these genes involved in the Notch pathway prompted us to investigate whether the in vitro pharmacological inhibition of Notch signalling could provoke similar effects as those observed in the incisors upon genetic epithelial Notch2 deletion. For this purpose, the LS8 dental epithelial cell line was used for the in vitro culture set-up. Cells were cultured either in the presence of 50 mM of the pan-Notch blocker CB103 [37] (treated group) or an equivalent volume of DMSO (control group) for 48 h (Figure 10a). The Alamar Blue viability assay has shown that the CB103-treated cultures were less dense and viable compared to the control cultures (Figure 10b–d). Upon culture, we performed RNA extraction and qRT-PCR analysis to first analyse the expression of downstream genes belonging to the Notch pathway. Results confirmed the effective inhibition of Notch signalling by showing a critical downregulation of the important Notch target (downstream) genes *Hes1*, *Hes2*, *Hes5*, *Hey1*, *Hey2 and HeyL* (Figure 10e).

We then sought to know if the in vitro Notch inhibition by CB103 also replicates the cell differentiation delay observed in the incisors of *Keratin14*^Cre/+^;*Notch2*^fl/fl^ mice. For this purpose, we first analysed the expression of the tight junction genes *Claudin1* and *Claudin5* (Figure 10f), and of the *Enam*, *Ambn*, *Amelx*, *Mmp20* and *Klk4* genes (Figure 10g) that are involved in amelogenesis. qRT-PCR analysis in CB103-treated LS8 cells demonstrated a marked downregulation in the expression of all these genes when compared to the DMSO-treated LS8 cells (Figure 10f,g).

Along with transcript analysis, we performed immunofluorescent staining against the Claudin1, Claudin5, Amelogenin, and Ameloblastin molecules. Our results demonstrated strong immunostaining for Claudin1 (Figure 10h) and Claudin5 (Figure 10i) in DMSO-treated cells, while the labelling was very faint, if not absent, in CB103-treated cells (Figure 10j,k). Similarly, in DMSO-treated cells, strong Ameloblastin (Figure 10k) and Amelogenin (Figure 10l) staining was observed. By contrast, in the CB103-treated cells, the Ameloblastin staining was absent (Figure 10n) and the Amelogenin labelling was considerably reduced (Figure 10o).

## 4. Discussion

The Notch signalling pathway plays a crucial role in regulating various cellular processes, such as cell fate specification, proliferation, and differentiation [2,7,8,40]. Previous studies on rodent teeth have demonstrated that inhibition of Notch activity leads to loss of intercellular communication and results in enamel defects [10,26]. These studies have identified the Notch1 receptor as a key regulator of the interaction between the stratum intermedium (si) cells and the ameloblasts and highlighted its importance in the homeostasis and regeneration of rodent incisors [10,20]. Upon incisors’ injury, the Notch1-expressing si cells can differentiate into ameloblasts [20]. Similarly, upon epithelial *Adam10* deletion, the *Notch1*-expressing cells of the si give rise to ameloblasts [10,41]. These data show that the dental epithelium possesses great plasticity and recovery mechanisms, often mediated through Notch signalling and the dynamic interplay between various cell populations. While the function of Notch1 in amelogenesis has already been established, the specific role of Notch2 in enamel formation remains largely unknown [10,42].

In the present study, we demonstrated that *Notch2*-expressing cells and their progeny contribute to the developing mouse incisors’ epithelium formation. Although *Notch1*-expressing cells and their progeny are mainly restricted to cells of the si layer, *Notch2*-positive cells are widely distributed to the outer enamel epithelium (oee), stellate reticulum (sr), and si layer of the incisor’s epithelium. These results indicate that *Notch2*-expressing cells and their progeny are important for incisors’ homeostasis and that their absence might affect the process of amelogenesis and lead to defective enamel. Indeed, our findings show that the epithelial Notch2 deletion delayed incisors’ eruption and affected dental epithelium organisation and enamel structure. These findings mirror significant features of amelogenesis imperfecta in humans, an inherited disorder characterised by dysfunctional enamel protein deposition and hypoplastic and/or hypocalcified enamel that affects 1:700 people worldwide [43]. Previous studies demonstrated that epithelial Adam10 loss or concurrent inactivation of Notch signalling by blocking antibodies results in incisors with enamel malformations. These enamel defects were more severe than those observed upon, suggesting that Notch2 is mostly involved in the fine-tuning of enamel’s structure [15,40]. Amelogenesis imperfecta is a developmental disorder most commonly associated with mutations in enamel-specific genes such as *Amelx*, *Ambn*, and *Enam* [44]. Our early stage knock-out model indicates that Notch2 deletion modifies the expression of these genes from the onset of enamel development. These findings underscore *Notch2* as a potential upstream regulator of enamel formation and suggest its possible involvement in human enamel disorders. It is important to note that the fundamental differences between the human and mouse dentition might influence how Notch activity impacts enamel biology. Nonetheless, our findings open new avenues for exploring epithelial signalling and contribute to the search for future therapeutic strategies in treating enamel pathologies.

Amelogenesis is a complex, stepwise process involving synthesis, secretion, and deposition of organic components to generate enamel [45]. The significant downregulation of enamel-specific genes, including *Enamelin* (*Enam*), *Ameloblastin* (*Ambn*), *Amelogenin* (*Amelx*), and *Kallikrein-4* (*Klk4*), indicates defects in ameloblast functionality. The transport and final deposition of the enamel matrix proteins at the apical end of the ameloblasts is realised through a vesicle-mediated transport mechanism [46]. The cytoplasmic vesicles fuse with the cellular membrane at the apical protrusions of the ameloblasts (Tomes’ processes), which are structures extended into the enamel-forming space, where the enamel proteins are secreted [46]. Findings from a previous study linked the inadequate deposition of Amelogenin from ameloblasts exhibiting short and irregular protrusions [47]. A similar effect correlating the unusual accumulation of enamel proteins to aberrant Tomes’ processes is also noticed in incisors upon *Notch2* deletion.

The defects in enamel protein secretion/deposition and enamel structure indicate further irregularities in the dental epithelium of the incisor before and during the initiation of enamel synthesis. Numerous studies have demonstrated that the integrity of cellular connections between the epithelial layers of the incisor is essential for the correct secretion of enamel-related proteins and the formation of enamel [26,27]. Such intercellular connections within the dental epithelium are established through junctional complexes [38,39,48,49,50]. Given that ameloblasts are in direct contact with the adjacent si layer, these cell-to-cell interactions can significantly impact the orientation and stability of ameloblasts, ultimately affecting their functionality. Notably, previous studies reported that decreased integrity of the si-ameloblast physical connections leads to severe enamel defects [10,26]. Our findings in the *Keratin14*^Cre/+^;*Notch2*^fl/fl^ incisors show disorganisation in all epithelial cell layers, including the ameloblast layer, thus indicating reduced cell-to-cell cohesion.

Previous studies have linked Notch signalling to cell junctions and their function in healthy and pathological tissues [8,51,52]. Notch2 dysfunction has been associated with epithelial disorders and pathologies, where defective stem cell regulation and alterations in tight junction proteins contribute to the progression of various diseases, including skin pathologies, metastatic cancers, inflammatory bowel disease, and Alagille syndrome [8,52,53,54,55]. In several of these severe clinical cases dental anomalies have also been noted, but remain poorly characterised, likely overshadowed by the prominent systemic symptoms [56,57]. Our data suggest that Notch2 plays an important and non-redundant role in enamel formation and epithelial organization, providing a mechanistic framework that may underlie enamel defects observed in these patients. Therefore, it would be interesting to investigate the extent of dental malformations in these cases to fully estimate the impact of Notch2-related pathologies. A decreased expression and abnormal distribution of the tight junction proteins Claudin1, Claudin5, and Claudin10 are observed in the epithelium of the mutant incisors. The loss of Claudin1 and Claudin5 from the apical part of ameloblasts may affect their structural orientation and polarity, thereby disrupting their efficiency in enamel deposition and subsequent maturation. The absence of Claudin1, Claudin5, and Claudin10 at the basal part of ameloblasts indicates defects in anchoring points between the ameloblasts and si cells that could also interfere with this process.

Epithelial tissue turnover is strongly connected to the balance between stem cell maintenance, proliferation and differentiation [58]. Sox2-positive cells significantly decreased in the cervical loop of the *Keratin14*^Cre/+^;*Notch2*^fl/fl^ incisors, thus suggesting the loss of a subpopulation of stem cells that co-express the *Notch2* and *Sox2* genes. Reduction of *Sox2*-expressing cells at the cervical loop is accompanied by an increased number of Ki67-positive cells and a spatial shift in ameloblastin expression, thus suggesting that Notch2 deletion attenuates dental epithelial turnover that probably leads to the delayed eruption of the mutant incisors. Interestingly, upregulation of other Notch molecules, including ligands and downstream effectors, was insufficient to fully rescue the defects caused in the organisation and structure of Notch2-deficient incisors. This suggests that Notch2 may have a non-redundant, context-specific role and the upregulation of other Notch pathway components may not fully substitute for Notch2 function in maintaining epithelial integrity. In contrast to our in vivo model, our in vitro system using CB103 broadly inhibits all canonical Notch signalling, eliminating the possibility of compensation from other receptors. Nonetheless, pan-Notch signalling inhibition produced similar changes in amelogenesis-related genes and tight junction markers, further highlighting the specific contribution of Notch2 to these pathways.

Given the broader implications of Notch2 in epithelial tissue function and disease, these results provide new insights into how Notch signalling ensures structural integrity and coordinated differentiation in renewing epithelia.

## 5. Conclusions

**Figure 11:**
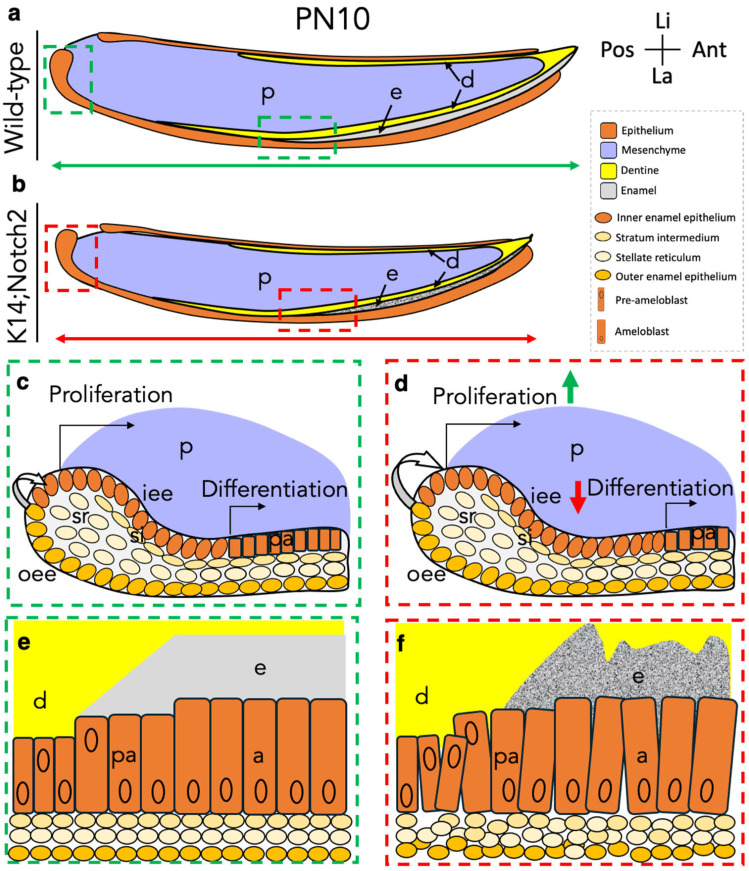
Schematic hypothetical model illustrating the impact of the epithelial Notch2 deletion during early postnatal incisor development. Comparison between the developing wild-type (**a**) and *Keratin14*^Cre/+^;*Notch2*^fl/fl^ incisors (**b**) showing a smaller size and defective enamel formation of the mutant incisors. Dashed boxes highlight the incisor’s areas affected upon Notch2 deletion. Higher magnification of the labial cervical loop area, the reservoir of dental epithelial stem cells, in the wild-type (**c**) and mutant incisors (**d**), indicating alterations in cell proliferation and differentiation in the *Keratin14*^Cre/+^;*Notch2*^fl/fl^ incisors. The dental epithelium consists of a core of stellate reticulum (sr) cells surrounded by inner epithelial cells (iee) and outer enamel epithelial cells (oee). Stratum intermedium (si) cells lie between the sr and iee layers. Slow-cycling cells of the oee and sr layers give rise to transit-amplifying progenitor cells (white arrow), which will exit the cell cycle and differentiate into pre-ameloblasts (black arrows). *Notch2*-expressing cells and their progenitors are located in the oee and sr layers. Deletion of Notch2 from the dental epithelium results in a decreased number of slow-cycling stem cells (white arrows in (**c**,**d**)), increased proliferation of the transit-amplifying progenitor cells (green arrow in (**d**)), and a delay in their differentiation into pre-ameloblasts (red arrow in (**d**)). Higher magnification of the enamel formation areas in the wild-type (**e**) and mutant incisors (**f**), highlighting the disorganisation of pre-ameloblasts/ameloblasts and defective enamel formation in the *Keratin14*^Cre/+^;*Notch2*^fl/fl^ incisors (**f**). Epithelial Notch2 deletion impairs intercellular connections and disrupts ameloblast polarisation, leading to defective enamel formation. Abbreviations: a, ameloblasts; ant, anterior; e, enamel; iee, inner enamel epithelium; la, labial; li, lingual; oee, outer enamel epithelium; p, pulp; pa, pre-ameloblasts; pn, postnatal day; si, stratum intermedium; sr, stellate reticulum.

In conclusion, our findings highlight the essential role of Notch2 in maintaining epithelial integrity and enamel formation during early postnatal incisor development. Epithelial Notch2 deletion leads to delayed incisor eruption, dental epithelial disorganisation, aberrant enamel-specific gene expression and protein distribution, and defective enamel formation. The observed enamel defects resemble features of amelogenesis imperfecta, suggesting a potential link between Notch signalling disruption and enamel pathogenesis. The present results further underscore the involvement of Notch2 in regulating epithelial cell differentiation and junctional integrity, revealing the crucial role of the Notch signalling pathway in dental tissue organisation and proper tooth development (Figure 11).

## Data Availability

The datasets used and/or analysed during the current study are available from the corresponding and first authors on reasonable request.

## Abbreviations

AMBN	Ameloblastin
AMELX	Amelogenin
BSA	Bovine Serum Albumin
DEJ	Confocal Laser Scanning Microscope
DMSO	Dimethyl sulfoxide
E	Embryonic Day
EDU	5-Ethynyl-2′-Deoxyuridine
ENAM	Enamelin
GFP	Green Fluorescent Protein
IEE	Inner Enamel Epithelium
IF	Immunofluorescence
IHC	Immunohistochemistry
KLK4	Kallikrein 4
KO	Knockout
laCL	Labial Cervical Loop
liCL	Lingual Cervical Loop
μm	Micron
MMP20	Matrix Metalloprotease 20
NICD	Notch Intracellular Domain
OEE	Outer Enamel Epithelium
PN	Postnatal Day
PCR	Polymerase Chain Reaction
qPCR	Quantitative Polymerase Chain Reaction
SI	Stratum Intermedium
SOX2	SRY-box transcription factor 2
SR	Stellate Reticulum
